# Isolated Unilateral Orbital Compression Syndrome in A 19-Year-Old Male With Homozygous Sickle Cell Disease

**DOI:** 10.7759/cureus.18545

**Published:** 2021-10-06

**Authors:** Fatema M Almukhtar, Fatema M Aljufairi

**Affiliations:** 1 Ophthalmology, Almosawi Specialist Center, Bilad Al Qadeem, BHR; 2 Ophthalmology, Salmaniya Medical Complex, Manama, BHR

**Keywords:** orbit, oculoplasty, optic nerve dysfunction, proptosis, orbital infarction, evacuation, sub-periosteal hematoma, sickle cell disease, orbital compression syndrome

## Abstract

This study aimed to report a rare case of a rapidly progressive isolated unilateral orbital compression syndrome in a male with homozygous sickle cell disease, who presented with proptosis and optic nerve dysfunction. He neither had long bone pain crisis nor fever at the time of presentation that was managed surgically to preserve vision.

Rapidly progressive left orbital swelling is observed in a 19-year-old homozygous sickle cell disease patient associated with severe pain, headache, and impaired vision. Computed tomography of the orbit confirmed the presence of a unilateral large superior sub-periosteal cystic mass. Surgical exploration via anterior orbitotomy revealed a large sub-periosteal hematoma occupying the superior orbit which was evacuated. The patient completely recovered within 14 days post-surgery and regained his vision.

Orbital involvement in sickle cell disease is rare, however, it can occur as a sequela of vaso-occlusive crisis and bone marrow infarctions leading to bleeding and sub-periosteal hematomas in the orbit. Prompt diagnosis and management of orbital compression syndrome are crucial to prevent permanent optic nerve damage. Hence, cautious evaluation and close monitoring are important, especially in cases where surgical evacuation is indicated for quick recovery and prevention of visual loss.

## Introduction

Sickle cell disease is a group of inherited monogenic hematological disorders that affect the red blood cells [1,2]. It is caused by the substitution of the amino acid valine for glutamic acid in the β-globin chain [3]. This mutation in gene coding creates inflexible sticky crescent-shaped RBCs; incapable to transverse the capillary bed, causing organs' ischemia-reperfusion injuries [4].

Sickle cell disease can be divided into five subtypes depending on the inherited genes from both parents [3]. The most common subtype worldwide is the hemoglobin SS, followed by hemoglobin SC disease and hemoglobin Sβ thalassemia. Hemoglobin SD and hemoglobin SE are the other subtypes that are less noticed [5].

Vaso-occlusive process in sickle cell disease comprises all the different systems of the body. People with sickle cell disease mainly experience anemia, bone pain, acute chest syndrome, ischemic stroke syndrome, and sudden death. Others can also suffer from chronic restrictive lung disease, pulmonary hypertension, dysrhythmias, hemorrhagic stroke, venous sinus thrombosis, silent cerebral infarction, cognitive impairment, avascular necrosis, leg ulceration, renal failure, nocturnal enuresis, priapism, cholelithiasis, or mesenteric vaso-occlusion [6]. 

Moreover, the eye also shows different sickling signs including corkscrew vessels and comma-shaped capillary segment involving the conjunctiva, iris ischemic atrophy, and cataract [7]. The posterior segment can exhibit non-proliferative changes such as tortuous veins, silver-wiring of arterioles, salmon patches, black sunbursts, macular depression sign, peripheral retinal holes, or angioid streaks, in addition to proliferative changes including peripheral arteriolar occlusion, arteriovenous anastomoses, sea-fan shaped neovascularization, auto-infarcted greyish fibrovascular lesions, vitreous hemorrhage, and retinal detachments either rhegmatogenous or tractional [8].

Although orbital involvement in sickle cell disease is rare, when it does occur, it could even be complicated by orbital compression syndrome; this develops when infarction of the microvasculature in the sphenoid bone marrow leads to inflammation and necrosis, which in turn cause subperiosteal hemorrhage [9]. This is either managed medically or surgically whenever there is optic nerve dysfunction or large hematomas [10]. In fact, 16 case reports exist in the literature describing orbital involvement in the last three decades and only four of them have been managed surgically [10,11]. In this study, we report a unilateral orbital compression syndrome in a homozygous sickle cell disease a 19-year-old man, in whom proptosis and eye pain were the only presenting symptoms.

## Case presentation

A 19-year-old man with sickle cell disease, cashier by profession, and a hookah smoker presented to the ophthalmic emergency department on June 25, 2021, with a one-day history of frontal headache followed by sudden left eye pain associated with increasing orbital swelling and decreasing vision. The patient denied any history of fever or bone pain at or prior to presentation. However, he reported a history of sudden hearing loss six years ago for which a cochlear implant surgery was performed.

**Figure 1:**
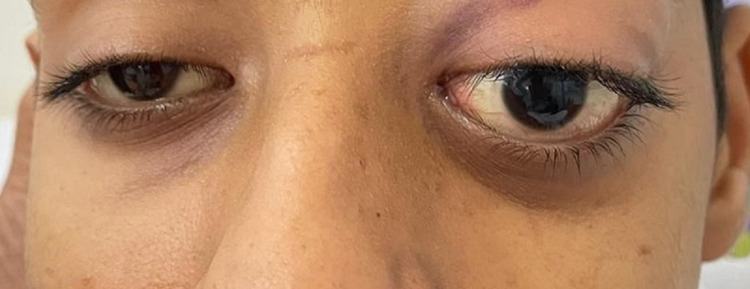
Photograph of both eyes demonstrating apparent left eye proptosis and dystopia.

**Figure 2:**
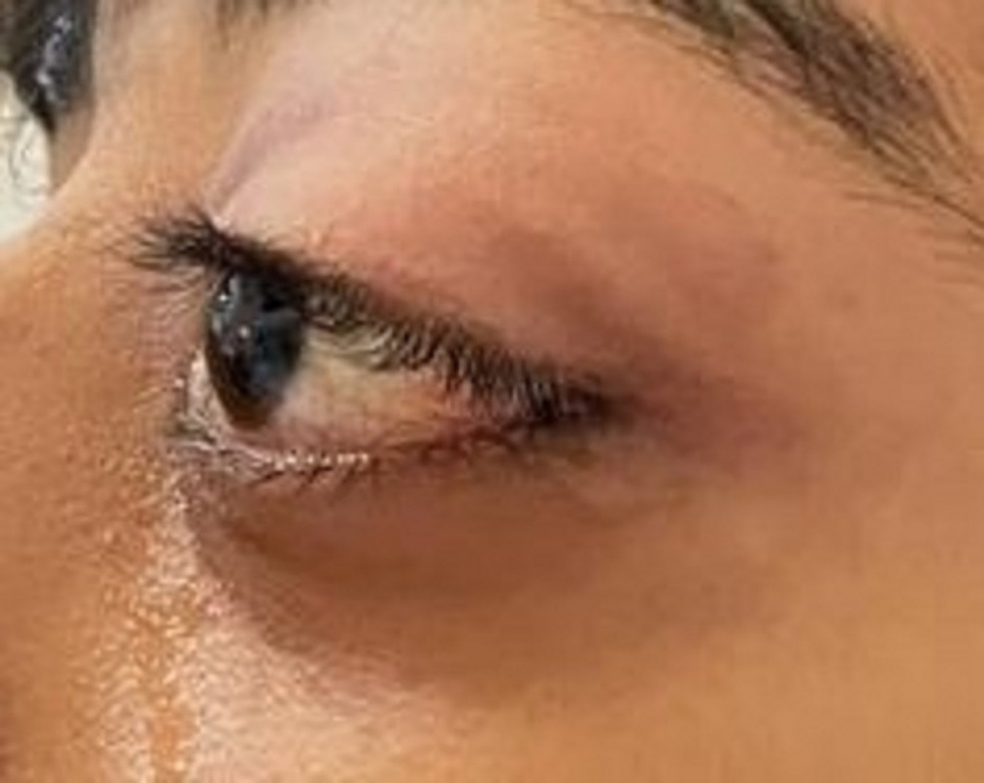
Side photograph of the left eye demonstrating clinically significant proptosis.

**Figure 3:**
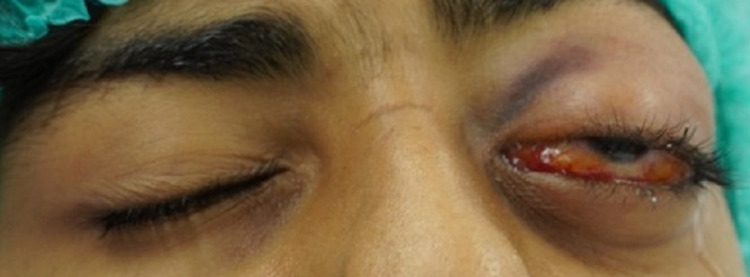
Photograph of both eyes showing left eye lagophthalmos (around 3 mm).

The patient was in severe pain but remained alert. His body temperature was 36.2°C, blood pressure was 126/90, heart rate was 88/min, and oxygen saturation was 98% on room air. Ocular examination showed a vision of 6/6 in the right eye, while examination of the left eye showed a vision of 1.5 m counting fingers, non-axial proptosis with a dystopia of 8 mm to the inferolateral side, vertical palpebral height was 16 mm, with periocular fullness, tenderness on palpation and resistance to retropulsion (Figures 1, 2). Moreover, extraocular muscles were restricted in all gazes with a 3-mm lagophthalmos (Figure 3).

Although posterior segment and optic disc examination appeared completely normal, the pupil assessment revealed relative afferent pupillary light reflex defect grade 3 with positive color desaturation.

Laboratory results showed white blood cell count was 3.78 x 10^9^/L (normal range: 3.6-9.6), hemoglobin level was 10.7 g/dL (normal range: 12.0-14.5), mean cell volume was 85.1 fL (normal range: 80.0-97.0), mean corpuscular hemoglobin was 27.8 pg (normal range: 27.0-33.0), and mean platelets volume was 10.1 fL (normal range: 8.0-11.5). Liver function test revealed a total bilirubin of 198 µmol/L (normal range: 5-21), direct bilirubin of 36 µmol/L (normal range: 0-5), indirect bilirubin of 162 µmol/L (normal: <18), and alkaline phosphatase of 315 U/L (normal range: 50-136). Coagulation profile was measured to be prothrombin time of 16.9 seconds (normal range: 10-14), international normalization ratio of 1.42 (normal range: 0.6-1.17), activated partial prothrombin time of 24.1 seconds (normal range: 28-43), and thrombin time of 13.1 seconds (normal range: 15.6-18.4). The erythrocyte sedimentation rate was 15 mm/h (normal < 20), and C-reactive protein was 151.22 mg/dL (normal range: 0-3). The kidney function test was normal. Hemoglobin electrophoresis showed sickle hemoglobin (HbS) 78.1%, fetal hemoglobin (HbF) 15.8%, and hemoglobin A2 (HbA2) 3.5%, which are compatible with homozygous sickle cell disease.

**Figure 4:**
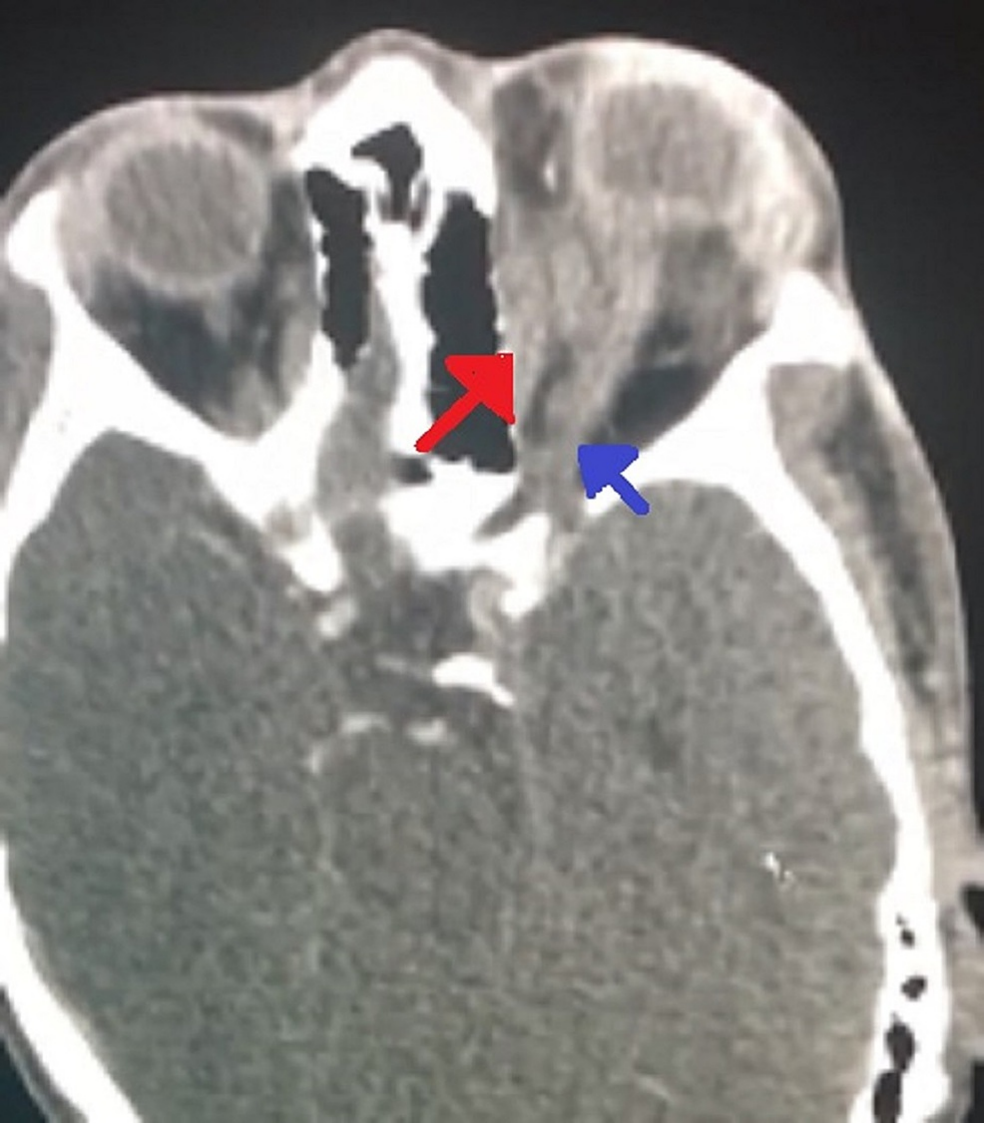
Axial computed tomography scan of both orbits showing left eye proptosis caused by nasal extraconal cystic mass (red arrow) and a kinked optic nerve (blue arrow).

**Figure 5:**
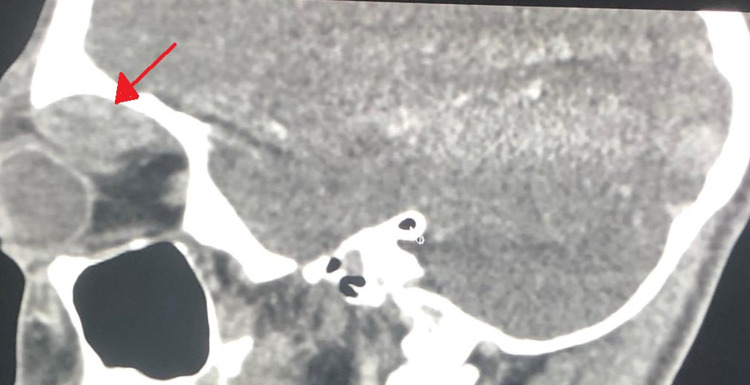
Sagittal computed tomography scan showing left superior extraconal cystic mass (red arrow) compressing the globe causing dystopia and proptosis.

Computed tomography scan of the orbit was obtained and revealed a large cystic extraconal mass lesion in the upper part of the left orbit, measuring 2.8 x 3.3 x 1.3 cm. The mass was compressing and displacing the globe and optic nerve infero-laterally, resulting in marked proptosis and orbital edema suggestive of sub-periosteal hematoma (Figures 4, 5).

The patient was started on intravenous methylprednisolone 1 g once daily along with intravenous Co-amoxiclav 1.2 g every 8 h. The decision was made to surgically intervene on the day of admission, so the patient was immediately prepped for surgical exploration under general anesthesia.

**Figure 6:**
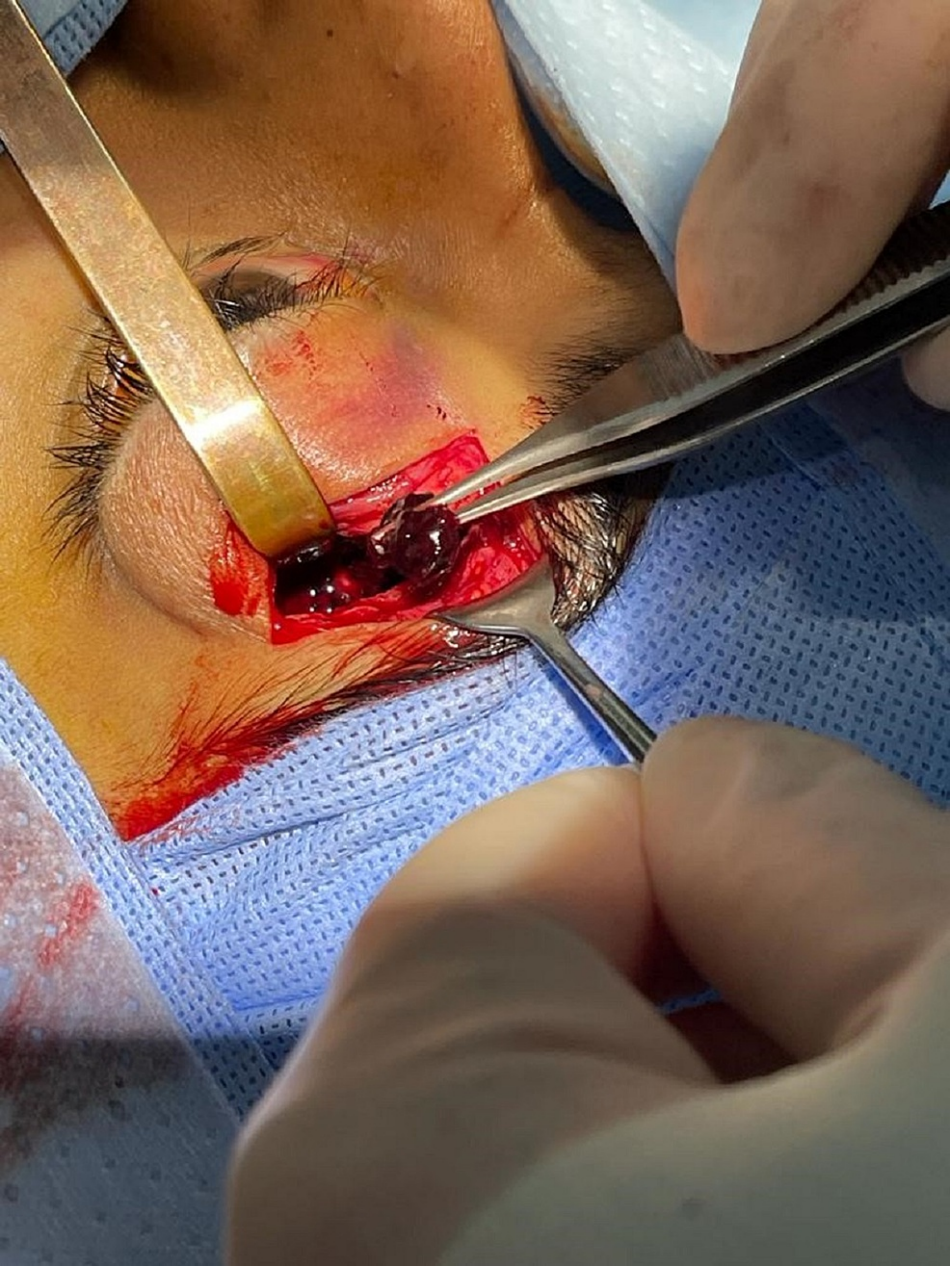
Intra-operative photograph showing the evacuated blood clot via sub-brow incision.

**Figure 7:**
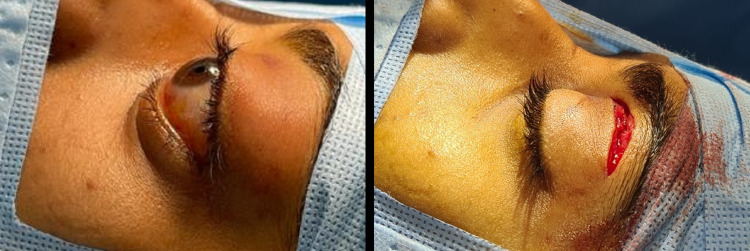
Pre-operative and post-operative photographs showing immediate resolution of proptosis after evacuation of hematoma.

A sub-brow approach was performed through a 2-cm incision. When periosteum was incised, a large sub-periosteal hematoma with blood clots was seen and entirely evacuated (Figure 6). Immediate improvement of proptosis was noticed on the table and the globe was back to normal position (Figure 7). Hemostasis was achieved and the incision was sutured in two layers with interrupted 6-0 vicryl sutures. The evacuated material was sent for histopathology and confirmed blood products.

**Figure 8:**
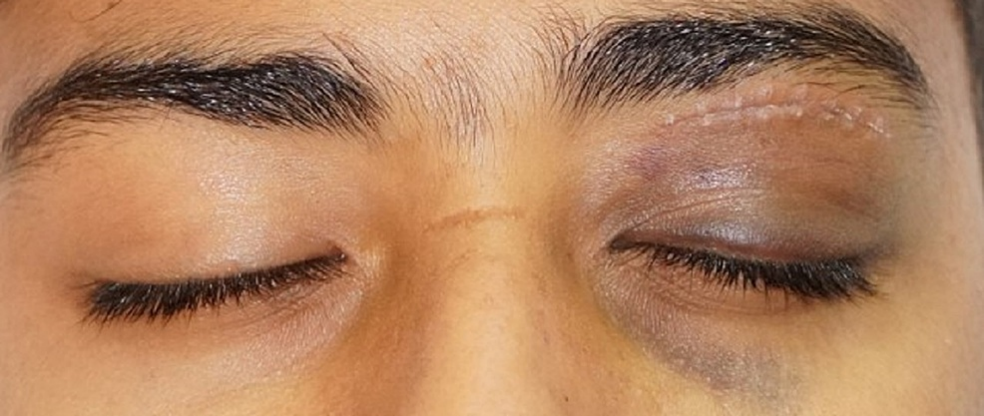
Two weeks post-operative photograph after removal of sutures showing clean wound with good healing and no lagophthalmos.

**Figure 9:**
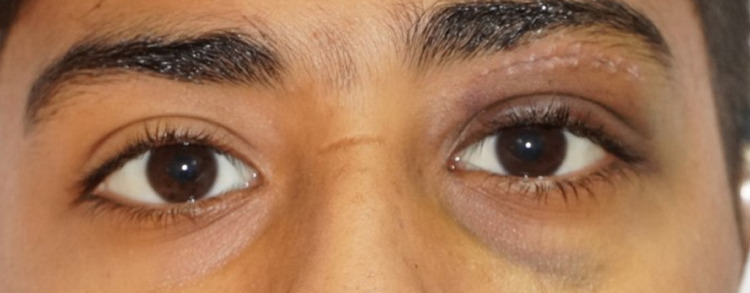
Two weeks post-operative photograph showing central light reflex in primary gaze with normal palpebral fissure height.

**Figure 10:**
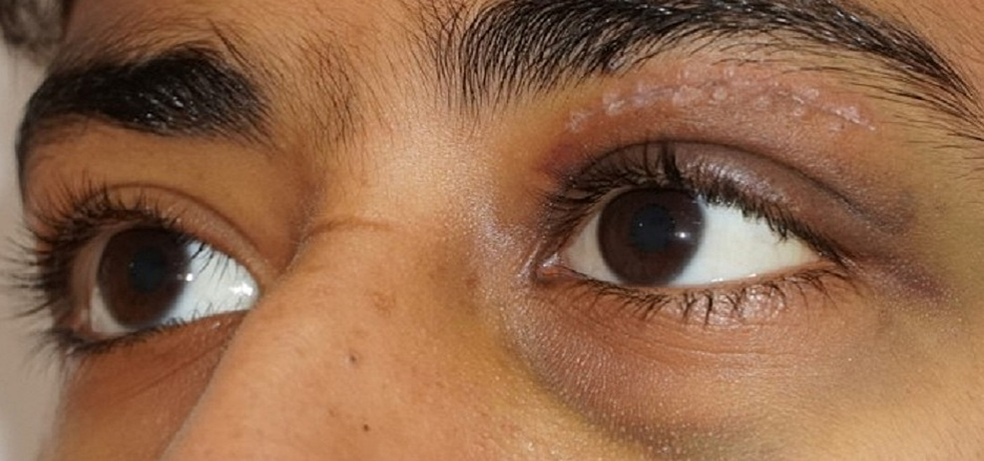
Two weeks post-operative photograph showing no apparent proptosis.

The patient had immediate resolution of proptosis, pain and visual acuity improved to 6/18 on the first post-operative day with mild lid edema. He regained full range of extraocular movements in all directions of gaze with no lagophthalmos. The patient received a total of three doses of daily intravenous methylprednisolone and was discharged home on oral antibiotics on the fourth post-operative day. He was advised of a follow-up visit to the outpatient department after one week. Two weeks later, the patient had a full recovery with the restoration of visual acuity to 6/6 in the affected eye (Figures 8-10).

## Discussion

Orbital compression syndrome rarely presents as a complication of sickle cell disease. To the best of our knowledge, 16 previous case reports exist describing orbital compression syndrome in the last three decades. All previous articles reported having either acute pain crisis preceding the orbital compression event, documented fever, or a rise of white blood cells on admission (Tables 1-3) [10-19].

**Table 1:** Summary of previous case reports (1/3) RE: right eye; LE: left eye; RAPD: relative afferent pupillary defect

Case report		Curran et al. [12]	Ganesh et al. [13]	Khouri et al. [11]	Procianoy et al. [14]
Year of publication		1997	2001	2002	2008
Age		8 years	6-15 years	11 years	11 years
Gene		Sickle β thalassemia	4 patients: Homozygous sickle cell disease; 1 patient: Sickle cell-β-thalassemia	Homozygous sickle cell disease	Not mentioned
Laterality		Bilateral	4 patients: Unilateral; 1 patient: Unilateral	Bilateral	Unilateral
History	Bone pain	Yes	Yes	Yes	No
	Fever	Yes	Yes	No	No
	Trauma	No	No	No	Yes, minor one
	Other	-	-	Cerebrovascular accident	-
WBCs		Increased	Not mentioned	Increased	Not mentioned
Optic nerve function		RE normal, LE: RAPD + Decrease in vision	Decrease in vision	No RAPD	Decrease vision RAPD
Treatment		Both eyes orbital decompression + intravenous analgesics, fluids, and antibiotics, and packed erythrocyte transfusion	intravenous fluids, analgesics, antibiotics and steroids, and exchange transfusion	Surgical Evacuation (misdiagnosed first as abscess)	Surgical intervention twice
Outcome		Regained vision	All regained vision 6/6	The swelling resolved	Regained vision

**Table 2:** Summary of previous case reports (2/3) URTI: upper respiratory tract infection; RAPD: relative afferent pupillary defect; NPL: no perception of light

Case report		Soko et al. [15]			Helen et al. [16]
Year of publication		2008			2013
Age		22 years	16 years	10 years	11 years
Gene		All homozygous sickle cell disease			Homozygous sickle cell disease
Laterality		All unilateral			Bilateral
History	Bone pain	No	No	Yes	Yes
	Fever	No	No	Not mentioned	Yes
	Trauma	No	No	No	No
	Other	-	URTI	-	-
WBCs		Increased	Not mentioned	Increased	Not mentioned
Optic nerve function		Decrease in vision + Compressive optic neuropathy	Normal	Decrease in vision + No RAPD	Loss of vision due to bilateral bullous retinal detachment
Treatment		All Intravenous antibiotics and steroid			Systemic and topical steroid, intravenous fluid, and analgesics
Outcome		Resolution of edema			Improved swelling but vision remained NPL

**Table 3:** Summary of previous case reports (3/3) RAPD: relative afferent pupillary defect; RE: right eye; LE: left eye; PL: perception of light

Case report		Yateem et al. [10]	Sundu et al. [17]	Alghamdi [18]	Onyeama and Jain [19]
Year of publication		2015	2017	2018	2020
Age		10 years	14 years	12 years	2 years
Gene		Sickle cell-β-thalassemia	Not mentioned	Sickle cell-β-thalassemia	Homozygous sickle cell disease
Laterality		Bilateral	Bilateral	Unilateral	Unilateral
History	Bone pain	Yes	Yes	Not mentioned	Not mentioned
	Fever	Yes	Not mentioned	Yes	No
	Trauma	No	No	No	No
	Other	-	-	-	-
WBCs		Normal	Not mentioned	Increased	Not mentioned
Optic nerve function		MRI showed small optic nerve, displaced with loss of its surrounding cerebrospinal fluid	Minimal decrease in vision + No RAPD	Decrease in vision + No RAPD	Not mentioned
Treatment		-Hydration, intravenous antibiotics, and steroid -Urgent drainage of sub-periosteal hematoma	Intravenous antibiotics and steroid	Intravenous antibiotics and steroid	Intravenous fluid, antibiotics, systemic anti-inflammatory agents, and opioid analgesia
Outcome		RE 6/6, LE PL + Gradual improvement with reduction in swelling	Totally recovered	Condition was stabilized, Nothing mentioned about vision	Dramatic improvement

Unlike the previous case reports, detailed history taking, vital signs checking, and complete blood count workup all turned to be unremarkable, and proptosis and eye pain were the only presenting symptoms. Although the patient and his parents denied any previous significant sickling attacks, the necessity of undergoing both ears cochlear implantation following sensorineural hearing loss six years prior to the presentation can be explained by having a sickle cell crisis at that time.

Moreover, only four of the previously reported cases have required surgical intervention, while the rest improved with conservative management. Our case sets a good example of early recognition and prompt surgical intervention which proved crucial in regaining vision in cases with optic nerve dysfunction.

## Conclusions

Orbital compression syndrome, although rare, can occur in patients with sickle cell disease. As shown in our case, prompt surgical management is crucial for successful resolution and recovery of vision, especially in those cases complicated by optic nerve dysfunction due to large sub-periosteal hematoma. We suggest that future studies should focus on comparing different sickle cell disease variants and their association with orbital compression syndrome occurs.
